# Comment on Ivanov et al. Ultra-Hypofractionated vs. Moderate Fractionated Whole Breast Three Dimensional Conformal Radiotherapy during the COVID-19 Pandemic. *Medicina* 2022, *58*, 745

**DOI:** 10.3390/medicina58091306

**Published:** 2022-09-19

**Authors:** Gianluca Ferini

**Affiliations:** REM Radioterapia Srl, Viagrande, 95029 Catania, Italy; gianluca.ferini@grupposamed.com; Tel.: +39-095-78-94-581

I read the paper by Ivanov et al. [1] recently published in *Medicina* and I would like to underline some critical issues, which could lead to a misinterpretation of the reported data by the readers. First of all, the insiders know that BED and EQD2 are not the same thing, but the authors incorrectly mention the BED alongside the definition of EQD2. Similarly, in the abstract and throughout the paper, there is confusion about median and mean doses. Moreover, the authors should have explained why they decided to include only invasive breast carcinomas, since even ductal carcinomas in situ benefit from adjuvant irradiation [2]. It is unclear how the authors evaluated *the signs of deterioration of heart and lung*. Now I come to the point of my great concern: the authors compared the absolute dose prescriptions of two very different schedules without scaling in EQD2 and, on the basis of these comparison results, they concluded that the ultrahypofractionated schedule could be less toxic than the fifteen fractions. Such an inference is wrong since the risk of adverse radiation events is strongly dependent not only on the total dose but also on each fraction dose size [3]. For example and for better clarity, when I along with other authors published some papers about the risk of radiation proctitis among prostate cancer patients [4,5,6], we referred to different dose constraints during the plan evaluation based on the diversity of the dose prescriptions. Again, a total dose prescription of 20 Gy is common for brain radiotherapy [7]; obviously, such a dose has a very different risk profile depending on whether it is delivered to the entire brain in five fractions of 4 Gy each (whole-brain radiation therapy) or in a single shot (20 Gy stereotactic radiosurgery) to a metastasis strictly close to the brainstem. Therefore, assuming that an ultrahypofractionated scheme may be less toxic than a longer one with a downsized dose per fraction only on the basis of a rough analysis exclusively comparing absolute values without proper conversion, is a dangerously misleading interpretative mistake. This issue is particularly important in the high-risk scenario investigated by the authors, especially if no specific heart-sparing ploys for patients with left-sided breast cancer are adopted [8,9]. In my opinion, the only relevant finding of the questioned article is about the lower rate of COVID-19 infection among the patients treated with the five fractions schedule, which supports again the convenience of ultrahypofractionated radiotherapy in times of the pandemic [10]. I invite the authors, and possibly also the academic editors, to discuss the main point raised here.
